# Long-term cardiac effects of modern treatment for Hodgkin’s lymphoma

**DOI:** 10.1186/s40959-024-00222-4

**Published:** 2024-04-04

**Authors:** Anders W Bjerring, Knut HB Smeland, Thomas Stokke, Kristina H Haugaa, Espen Holte, Assami Rösner, Cecilie E Kiserud, Thor Edvardsen, Sebastian Imre Sarvari

**Affiliations:** 1 ProCardio Center for Innovation, Department of Cardiology, Oslo University Hospital, Rikshospitalet, Oslo N-0027 Norway; 2 Faculty of Medicine, University of Oslo, Oslo, Norway; 3 National advisory unit for late effects after cancer, Department of Oncology, Oslo University hospital, Oslo, Norway; 4 Department of Circulation and Medical Imaging, Clinic of Cardiology, St. Olavs University Hospital, Norwegian University of Science and Technology, Trondheim, Norway; 5 Cardiological Department, University Hospital North Norway, Tromsø, Norway; 6 Institute of Clinical Medicine, UiT the Arctic University of Norway, Tromsø, Norway

**Keywords:** Cancer survivorship, Hodgkin’s lymphoma, Echocardiography, Cardiotoxicity

## Abstract

**Background:**

Hodgkin’s lymphoma (HL) is a hematological malignancy that affects both children and young adults. Traditional treatment is associated with a life-time prevalence of cardiac disease exceeding 50%. In the late 1990s protocols were modified to reduce cancer therapy-related adverse cardiac effects. This study aimed to assess the long-term impact of advances in treatment protocols on the cardiac health of HL survivors (HLS).

**Methods:**

HLS (*n* = 246) treated between 1997 and 2007 with anthracycline-based chemotherapy in three centers in Norway were included. Of these, 132 (53%) had also received mediastinal radiotherapy. HLS were compared to controls (*n* = 58) recruited from the general population and matched for sex, age, smoking status, and heredity for coronary artery disease. All subjects underwent echocardiography, clinical assessment, and blood sampling.

**Results:**

The HLS were 46 ± 9 years old and had been treated 17 ± 3 years before inclusion in the study. There was no significant difference between HLS and controls in ejection fraction (EF) (58%±5 vs. 59%±4, *p* = 0.08) or prevalence of heart failure. HLS treated with both anthracyclines and mediastinal radiotherapy (AC + MRT) had slightly worse left ventricular global longitudinal strain than controls (-19.3 ± 2.5% vs. -20.8 ± 2.0%, *p* < 0.001), but those treated with only anthracyclines did not. HLS treated with AC + MRT had a higher prevalence of valve disease than those treated only with anthracyclines (12% vs. 4%, *p* < 0.05).

**Conclusions:**

HLS treated with anthracyclines after the late 1990s have similar cardiac function and morphology as age-matched controls, apart from higher rates of valvular disease in those who also underwent mediastinal radiotherapy.

## Background

Hodgkin’s lymphoma (HL) is a hematological malignancy that affects both children and young adults. Modern treatments of HL have greatly improved outcomes and most patients are now being cured, with 5-year survival following multi-agent chemotherapy with or without radiotherapy exceeding 80% [1].

While the treatment of HL at first glance seem like an unabridged success, there has been a cost in terms of increased overall late mortality, primarily through secondary cancer and cardiovascular disease (CVD) [2]. Damage to the cardiomyocytes brough on by antineoplastic therapy, dubbed cardiotoxicity, has been a major driver of morbidity and mortality in the treatment of HL. In all age groups, but particularly in younger patients, the rate of heart failure is substantial. In a recent meta-study, patients treated for HL had a 7-fold increase in risk of dying from CVD and those treated before the age of 21 had a 13-fold increase in risk [3]. Of 27 studies included in this meta-study, only five had included patients treated after 1997, and even in those the majority of patients included were treated prior to 1997.

In the 1980s and early 1990s, the treatment protocol for HL involved both the use of cardiotoxic anthracyclines and extended field radiation, such as mantle field, for many patients. This combination caused a dramatic rise in cardiovascular morbidity and mortality observed in HL survivors (HLS) [1]. Beginning in the late 1990s, treatment strategies shifted to minimize the amount of radiation delivered to cardiac tissue, both through reduced radiation doses and through more targeted delivery [4–6].

Although the changes have likely reduced mortality and morbidity in survivors, there is a paucity of studies evaluating cardiac function in HLS treated with modern protocols. As chemotherapy- and radiation-induced adverse cardiac effects often take years to develop into clinical heart disease, a comprehensive evaluation of late effects has simply not been viable until now.

Understanding the long-term risk of cardiac disease in cancer survivors is essential for making accurate and appropriate guidelines that provide clinicians with the proper tools for adequate long-term management of cardiovascular risk. Both the underestimation and overestimation of actual risk can adversely affect cancer survivors.

The aim of this study is to describe the long-term effects of modern treatment protocols for HL on cardiac function and morphology, using state-of-the-art echocardiographic methodology, including 3D and strain-measurements. We hypothesize that changes in treatment protocols have had a beneficial effect on long-term cardiovascular function but have not eliminated the issue.

## Methods

Written informed consent was given by all study participants. The study complies with the Declaration of Helsinki and was approved by the Regional Committee for Medical Research Ethics (ref. 2016/2311).

**Fig. 1:**
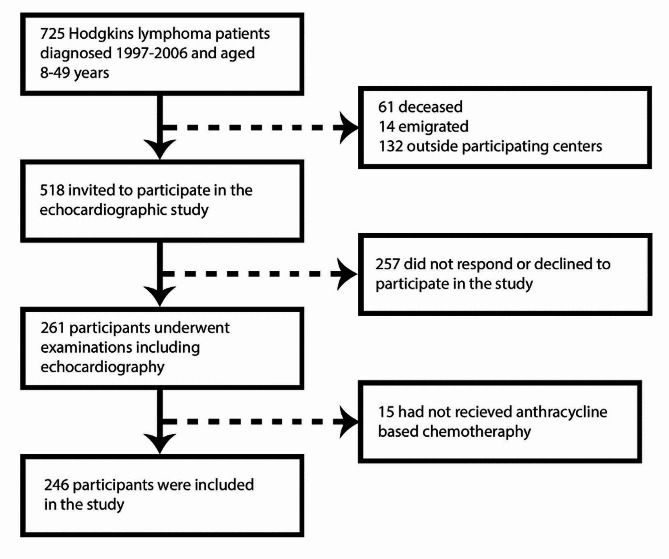
Flowchart of patient recruitment and selection

All patients diagnosed with HL at age 8–49 years between 1997 and 2007 in Health region South-East, Mid and North Norway, were identified by the Cancer Registry of Norway, and patients alive 31 December 2016 were invited to take part in the study (Fig. 1). HLS that had never received anthracyclines were excluded from our study. Participating HLS underwent a comprehensive clinical examination between 2018 and 2019, including blood samples, blood pressure measurements, echocardiography, and completed a questionnaire on cardiac risk factors, heart disease and current medications.

From 2019 to 2021, a control group was recruited from a database set up from the “Lier registry”, a population health study in conjunction with the Norwegian municipality of Lier [7]. The control group was matched for sex, age, smoking status and heredity for coronary artery disease.

### Diagnostic and treatment data

Patients with classical HL in stage I and II A underwent 2 to 4 courses of ABVD (doxorubicin, bleomycin, vinblastine and dacarbazine) followed by modified involved-field radiotherapy 30 to 35 Gy. Nodular lymphocyte-predominant HL in stages I and IIA were treated with 30 Gy involved-field radiotherapy. Most HL patients with disease in stages IIB to IV received 6 to 8 courses of ABVD, although selected patients received from 1999 onward 6 to 8 courses of BEACOPP (bleomcyin, etoposide, doxorubicin, cyclophosphamide, vincristine, prednisone, and procarbazine) [8]. Details on lymphoma diagnosis and treatment were obtained from medical charts and the clinical lymphoma database at Oslo University Hospital. For each HLS planned total cumulative dose of anthracycline (doxorubicin) was registered. Patients with relapse or progression after first line therapy were usually treated with high-dose therapy autologous stem cell support after salvage chemotherapy, most commonly IGEV (ifosfamide, gemcitabine and vinrelbine) or DHAP (dexamamethasone, cytarabine and cisplatin).

### Blood sampling, blood pressure and definitions

Fasting morning blood samples were obtained from all participants and analyzed for hemoglobin (Hb), glycated hemoglobin (HbA1c), N-terminal pro-brain natriuretic peptide (NT-proBNP), low-density lipoprotein (LDL), high-density lipoprotein (HDL), total cholesterol, triglycerides, creatinine, and C-reactive protein (CRP). The samples were analyzed using Cobas 6000 and 8000 (Roche Diagnostics, Mannheim, Germany).

Blood pressure was measured in the supine position. BSA and BMI was calculated from self-reported weight and height. Obesity and metabolic syndrome were defined in accordance with guidelines from the American Heart Association [9]. Hypertension was defined as either the use of antihypertensive medication or a systolic blood pressure ≥ 140 mmHg or a diastolic blood pressure ≥ 90 mmHg. Hypercholesterolemia was defined as either a total cholesterol of > 7 mmol/L or LDL ≥ 3.6mmol/L or the use of lipid-lowering medication [10]. All participants were assessed for cardiovascular risk using the Framingham Risk Score for Hard Coronary Heart Disease [11].

### Transthoracic echocardiography

All participants underwent an echocardiographic study (Vivid E95, GE, Vingmed, Horten, Norway). Echocardiographic images, including 3D images, were acquired from standard views. Analysis was performed post-hoc by a single observer using dedicated software (EchoPac, GE, Vingmed). All measurements were performed in accordance with recommendations from the European Association of Cardiovascular Imaging (EACVI) [12, 13].

Heart failure with reduced ejection fraction (HFrEF) was defined as an ejection fraction of ≤ 40% and symptoms of heart failure [14]. Heart failure with preserved ejection fraction (HFpEF) was defined as a Heart Failure Association Pre-test assessment, Echocardiography & natriuretic peptide, Functional testing, Final etiology (HFA-PEFF) score equal or greater than five, in accordance with the 2019 Heart Failure Association/European Society of Cardiology consensus recommendation [15].

Diastolic parameters were assessed by two-dimensional echocardiography (2DE). Diastolic function was assessed using left atrial (LA) volume, doppler derived velocities across the mitral valve, tricuspid regurgitation velocity and tissue doppler velocities. Diastolic dysfunction was considered present if more than half of the following criteria were met: average E/e’ >14, septal e’ <7 cm/s or lateral e’ <10 cm/s, TR velocity > 2.8 m/s, LA indexed volume > 34 ml/m^2^ [13]. All volume and areal measurements were indexed to body surface area (BSA).

2D speckle-tracking strain analysis Was used to assess global longitudinal strain (GLS), which was calculated from peak systolic longitudinal strain in 16 segments using standard views. Low GLS was defined as a GLS >-16.0, normal GLS was defined as GLS <-18.0, while GLS in between was defined as borderline [16]. The frame rate was 59 ± 7 Hz.

Valve pathology was considered present and significant when the patient had at least one of the following: at least moderate mitral valve insufficiency, at least moderate aortic valve insufficiency, any aortic valve stenosis, or any mitral valve stenosis. Valve disease was classified according to guidelines [17].

### Statistical analysis

Analyses were carried out using Stata 17.0 (StataCorp LLC, Texas, USA) and SPSS 28.0 (IBM SPSS Statistics, NY, USA). Data were presented as mean ± SD, median and interquartile range or numbers and percentages. Comparisons between two groups were performed using the χ2-test for categorical variables, the students t-test for continuous parametric parameters and the Mann-Whitney U test for continuous, non-parametric parameters. Comparison between three groups were performed using one-way ANOVA for continual, parametric data and Kruskal-Wallis one-way ANOVA for continual, non-parametric data. Comparison between individual groups in the ANOVA analysis were performed with the Bonferroni correction for multiple comparisons. HFrEF, HFpEF, significant valve disease and diastolic dysfunction were pre-determined important dichotomous outcomes and analyzed by logistic regression adjusted for sex, age, and treatment center. GLS, EF and TAPSE were pre-determined important echocardiographic parameters and analyzed by linear regression adjusted for sex, age, and treatment center. Correlations between these parameters and total anthracycline dose, dose of mediastinal radiation and history of autologous stem cell transplantation were analyzed by means of linear regression adjusted for sex, age, and treatment center, both in isolation and in an integrated regression. Two-sided p-values < 0.05 were considered significant. Reproducibility was expressed as intraclass correlation coefficient.

## Results

**Fig. 2:**
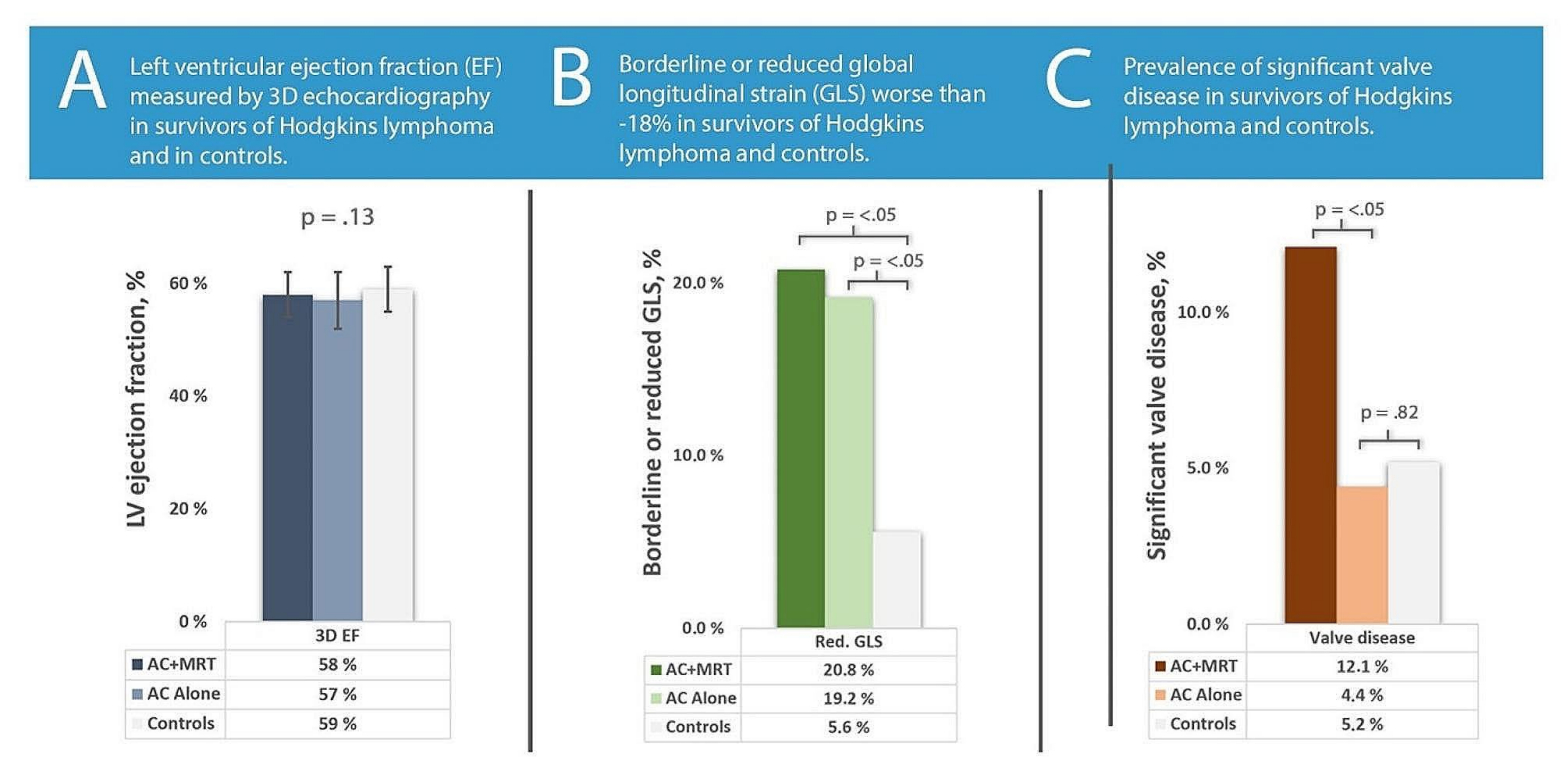
Comparison of Echocardiographic Parameters. (**A**)There was no difference in ejection fraction between study groups. (**B**) Hodgkin’s lymphoma survivors had a higher prevalence of borderline low or low global longitudinal strain. (**C**) Patients receiving both anthracycline-based chemotherapy and mediastinal radiotherapy had a higher prevalence of valve disease. P-values are derived from simple one-way ANOVA for continuous variables and χ2-test for categorical variables. *AC, Anthracycline-based chemotherapy; AC + MRT, Anthracycline-based chemotherapy and mediastinal radiotherapy; EF, ejection fraction; GLS, global longitudinal strain; LV, left ventricle*

A total of 246 patients with HLS were included from three cancer centers (169 from Oslo University Hospital, 47 from St. Olav Hospital and 30 from the University Hospital of Northern Norway) (Fig. 2). Attrition analysis on gender, age, age at diagnosis and time since diagnosis was performed between survivors who accepted the study invitation and 268 who did not. Four HLS were excluded from the attrition analysis due to lack of data. Those who accepted the study invitation were older (45.6 years vs. 43.7 years, *p* < 0.05) and more likely to be female (48.1% vs. 34%, *p* < 0.01). There were no differences with regards to age at diagnosis or time since diagnosis.

Out of the 246 HLS patients, 132 (54%) had undergone both anthracycline-based chemotherapy (AC) and mediastinal radiation therapy (MRT) (AC + MRT group), while 114 (46%) had only received anthracycline-based chemotherapy (AC group). Lymphoma stage and treatment regimens are summarized in Table 1.

**Table 1:** Classification and treatment in HL survivors

	AC + MRT (*n* = 132)	AC alone (*n* = 114)	p-value
**Histology and stage**			
Age at diagnosis, years	28 ± 9	30 ± 10	0.09
Time from diagnosis to echocardiography, years	17.3 ± 2.8	16.2 ± 2.8	**< 0.001**
Classical Hodgkin’s Lymphoma	131 (99)	100 (88)	**< 0.001**
Nodular lymphocyte-predominant Hodgkin lymphoma	1 (1)	13 (11)	**-**
Stage I-IIA	83 (63)	60 (53)	0.10
Stage IIb-IV	49 (37)	54 (47)	**-**
Relapsing disease	23 (17)	14 (12)	0.26
**Treatment**			
Cumulative anthracycline dose, mg/m2	200 (200–400)	220 (155–400)	0.81
Cumulative MRT dose, Gy	30 (30–35)	-	**-**
Vincristine/vinblastine	132 (100)	114 (100)	-
High-dose therapy with autologous stem cell support	20 (18)	12 (12)	0.24

Data expressed as n (%) for categorical parameters, mean ± SD for continuous, parametric parameters, and median (IQR) for continuous non-parametric parameters. P-value derived from the χ2-test for categorical variables, the students t-test for continuous parametric parameters and the Mann-Whitney U test for continuous, non-parametric parameters. BEP, bleomycin, etoposide, cisplatin; CBCT, cisplatin-based chemotherapy; CVB, cisplatin, vinblastine, bleomycin; EP, etoposide, cisplatin; MRT, mediastinal radio therapy

The observation time from treatment to echocardiography was 16.6 ± 2.9 years for all HLS. The AC + MRT group had a slightly longer observation time than the AC group (17.1 ± 2.8 years vs. 15.9 ± 2.9 years, *p* < 0.001). The HLS had received a median cumulative anthracycline dose of 200 mg/kg (IQR 180–400 mg/kg). Participants were compared to a group of 58 controls. Thirty-seven participants had relapsing disease and received repeated treatment. These received a higher median cumulative dose of anthracyclines than those without relapsing disease (335 mg/kg (IQR 200–400 mg/kg) vs. 200 mg/kg (IQR 160–400 mg/kg), *p* < 0.001).

### Risk factors and biochemistry

Basic characteristics, risk factors for coronary artery disease and biochemistry are summarized in Table 2.

**Table 2:** Basic characteristics, risk factors for CAD and laboratory measurements

	AC + MRT (*n* = 132)	AC alone (*n* = 114)	Controls (*n* = 58)	p-value (ANOVA)	p-value AC + MRT vs. AC alone	p-value AC + MRT vs. controls	p-value AC alone vs. controls
**Basic characteristics and risk factors**							
Age, years	46 ± 9	46 ± 10	47 ± 9	0.75	1.00	1.00	1.00
Males, n (%)	**50 (37)**	**76 (65)**	**31 (53)**	**< 0.001**	**< 0.001**	0.11	0.43
Body mass index, kg/m^2^	26.5 ± 5.3	26.6 ± 3.7	26.5 ± 4.9	1.00	1.00	1.00	1.00
Smoking (current or prior), n (%)	**42 (31)**	**59 (50)**	**21 (36)**	**0.008**	**0.006**	1.00	0.26
Systolic blood pressure, mmHg	131 ± 16	134 ± 17	134 ± 15	0.45	0.88	0.88	0.88
Diastolic blood pressure, mmHg	82 ± 11	84 ± 11	82 ± 9	0.51	1.00	1.00	0.79
High blood pressure, n (%)	56 (44)	48 (43)	21 (38)	0.70	1.00	1.00	1.00
* High measured BP*	49 (38)	43 (38)	20 (36)	0.94	1.00	1.00	1.00
* Antihypertensive med.*	23 (17)	16 (14)	4 (7)	0.20	1.00	0.23	0.66
High cholesterol, n (%)	39 (36)	46 (49)	19 (35)	0.11	0.16	1.00	0.30
* High measured chol.*	**28 (26)**	**40 (43)**	**20 (36)**	**0.048**	**0.044**	0.58	1.00
* Lipid-lowering med.*	**15 (11)**	**11 (9)**	**0 (0)**	**0.034**	1.00	**0.029**	0.16
Obesity, n (%)	26 (20)	24 (22)	12 (21)	0.93	1.00	1.00	1.00
Diabetes mellitus, n (%)	**3 (2)**	**8 (7)**	**0 (0)**	**0.038**	0.15	1.00	0.06
Established CAD, n (%)	9 (7)	4 (4)	1 (2)	0.22	0.64	0.34	1.00
* Prior myocardial inf.*	5 (4)	4 (4)	0 (0)	0.34	1.00	0.48	0.62
*Angina pectoris*	6 (5)	4 (4)	1 (2)	0.61	1.00	0.97	1.00
Framingham risk score, % (IQR)	**1.1 (0.4–3.4)**	**3.7 (0.8–11.9)**	**3.2 (1.0–8.0)**	**< 0.001**	**0.012**	**< 0.001**	1.00
Cardioprotective medication, n (%)	28 (21)	21 (18)	4 (7)	0.06	1.00	0.06	0.20
**Laboratory measurements**							
Total cholesterol, mmol/L	4.9 ± 0.9	5.0 ± 1.0	5.0 ± 0.8	0.43	1.00	0.65	1.00
Low density lipoprotein, mmol/L	3.1 ± 0.9	3.3 ± 1.0	3.2 ± 0.7	0.25	0.29	1.00	1.00
High density lipoprotein, mmol/L	1.5 ± 0.4	1.3 ± 0.4	1.4 ± 0.4	0.10	0.09	1.00	1.00
Triglycerides, mmol/L	1.3 ± 1.3	1.6 ± 1.1	1.3 ± 0.8	0.21	0.30	1.00	0.57
High-sensitive CRP, mg/L (IQR)	**0.8 (0–3.6)**	**0.0 (0–1.7)**	**0.8 (0.8–2.1)**	**0.035**	**0.034**	0.57	**0.025**
Pro-BNP, µmol/L (IQR)	**70 (27–124)**	**< 50 (0–63)**	**< 50 (0–78)**	**< 0.001**	**< 0.001**	**< 0.001**	0.81
HbA1c, %	36 ± 6	36 ± 8	34 ± 3	0.11	1.00	0.12	0.21
Creatinine,	**70 ± 15**	**72 ± 12**	**65 ± 9**	**< 0.05**	1.00	0.15	**0.039**
Hemoglobin, mg/dl	**13.7 ± 1.2**	**14.3 ± 1.2**	**13.3 ± 0.8**	**< 0.001**	**< 0.001**	0.41	**< 0.001**

Data expressed as mean ± SD for parametric data and median (IQR) for non-parametric data. P-values derived from simple one-way ANOVA for continual, parametric data and Kruskal-Wallis one-way ANOVA for continual, non-parametric data. Comparison between individual groups performed with the Bonferonni correction for multiple comparisons. Framingham risk score is the 10-year risk of CAD calculated using the Framingham Risk Score for Hard Coronary Heart Disease. Cardioprotective medication is defined as using at least one of the following: ACE-inhibitors, ARBs, beta-blockers, statins or ASA

HLS in the AC group were more likely to be male and to be current or former smokers. They were also more likely to have a high measured cholesterol than the AC + MRT group, but the difference in dyslipidemia was no longer significant when the use of lipid-lowering medication was included. Compared to controls, HLS were more likely to use lipid-lowering medication and to suffer from diabetes mellitus. Patients in the AC + MRT had a lower 10-year risk of CAD than both the CT-group and the controls, according to the Framingham Risk Score for Hard Coronary Heart Disease. There were no differences between any of the groups regarding hypertension or obesity.

NT-ProBNP was on average higher in the HLS than controls, and higher in the AC + MRT group than in the AC group. The AC group had higher hemoglobin levels than both the AC + MRT group and controls. While the ANOVA analysis showed a significant difference in CRP between groups, no specific groups comparisons were significant when adjusted for multiple comparisons.

### Cardiac morphology and function

Echocardiographic parameters are summarized in Table 3. HLS in the AC + MRT group had smaller left ventricle (LV) dimensions and mass than both the AC group and the controls. There were no significant differences in morphology between the AC groups and the controls.

**Table 3:** Echocardiographic data

	AC + MRT (*n* = 132)	AC alone (*n* = 114)	Controls (*n* = 58)	p-value (ANOVA)	p-value AC + MRT vs. AC alone	p-value AC + MRT vs. controls	p-value AC alone vs. controls
**Morphology**							
LA volume, mL/m^2^	**25 ± 6**	**27 ± 8**	**29 ± 7**	**0.016**	0.56	**0.012**	0.31
IVSd, mm	8.8 ± 2.0	9.1 ± 1.8	9.2 ± 1.8	0.33	0.74	0.57	1.00
LVIDd, mm	**47 ± 6**	**50 ± 5**	**49 ± 6**	**< 0.001**	**< 0.001**	**0.027**	0.92
LVPWd, mm	**7.8 ± 1.4**	**8.2 ± 1.3**	**8.3 ± 1.4**	**0.012**	**0.043**	**0.035**	1.00
LV Mass, g/m^2^	**67 ± 17**	**76 ± 18**	**76 ± 18**	**0.002**	**0.004**	**0.013**	1.00
3D LV EDV, mL/m^2^	**60 ± 9**	**64 ± 10**	**65 ± 10**	**0.012**	0.09	**0.015**	1.00
3D LV ESV, mL/m^2^	25 ± 6	28 ± 6	27 ± 5	0.06	0.06	0.45	1.00
RA area, cm^2^/m^2^	16 ± 3	17 ± 3	17 ± 3	0.06	0.15	0.14	1.00
RV end-diastolic area, cm^2^/m^2^	9.3 ± 1.5	9.6 ± 1.7	9.9 ± 1.6	0.18	0.05	0.34	1.00
RV end-systolic area, cm^2^/m^2^	5.6 ± 1.1	6.0 ± 1.1	5.8 ± 1.0	0.11	**0.020**	0.85	0.29
**Systolic function**							
3D LV EF, %	58 ± 5	57 ± 5	59 ± 4	0.13	1.00	0.32	0.15
3D LV SI, mL/m^2^	**35 ± 5**	**36 ± 6**	**39 ± 6**	**0.004**	0.49	**0.002**	0.14
LV GLS, %	**19.3 ± 2.5**	**20.2 ± 2.6**	**20.8 ± 2.0**	**< 0.001**	**0.034**	< **0.001**	0.34
RV FAC, %	40 ± 7	39 ± 6	41 ± 7	0.28	0.81	1.00	0.39
TAPSE, mm	**22 ± 3**	**22 ± 3**	**24 ± 3**	**0.001**	1.00	**0.002**	**0.004**
**Diastolic function**							
Mitral E velocity, cm/sec	67 ± 18	63 ± 15	65 ± 14	0.13	0.13	1.00	1.00
Mitral A velocity, cm/sec	**60 ± 16**	**54 ± 14**	**51 ± 15**	**< 0.001**	0.18	**0.001**	0.56
Mitral E/A-ratio	**1.2 ± 0.4**	**1.2 ± 0.4**	**1.4 ± 0.4**	**0.009**	0.92	**0.007**	0.08
Mitral DT, ms	**175 ± 47**	**187 ± 52**	**161 ± 46**	**0.005**	0.22	0.22	0.004
E/e’-ratio	7.7 ± 2.4	7.1 ± 2.8	6.9 ± 1.8	0.06	0.20	0.11	1.00
TR velocity, m/s	23 ± 3	23 ± 3	23 ± 2	0.99	1.00	1.00	1.00

Data expressed as mean ± SD for parametric data and median (IQR) for non-parametric data. P-values derived from simple one-way ANOVA for continual, parametric data and Kruskal-Wallis one-way ANOVA for continual, non-parametric data. Comparison between individual groups performed with the Bonferonni correction for multiple comparisons

Figure 2 summarizes EF, prevalence of borderline or reduced GLS and prevalence of significant valve disease in both treatment groups and in controls.

While average GLS did not differ between the AC group and controls, the AC + MRT group had worse GLS than both the AC group and the controls. Both groups of HLS had lower TAPSE than controls, but not worse RV FAC. EF did not differ between groups.

Three controls (5.6%), 20 in the AC group (19.2%) and 21 in the AC + MRT group (20.8%) had borderline or impaired GLS (GLS >-18.0%). There was no difference between treatment groups, but compared to controls, HLS had an OR for borderline or impaired GLS of 4.4 (1.3–14.9, *p* < 0.05) after adjusting for sex and age.

Three (3%) HLS in the AC group, none in the AC + MRT group and 1 (2%) control fulfilled the criteria for HFrEF. Echocardiographic criteria for diastolic dysfunction were fulfilled in 6 (6.3%) in the AC + MRT group, 6 (7.2%) in the AC group and in 6 (10.9%) controls. Ten participants fulfilled the criteria for HFpEF, 7 (5%) in the AC + MRT group, 3 (3%) in the AC group and none in the control group. There were too few HFrEF events for significance testing, while simple non-adjusted logistic regression remained non-significant for intergroup differences for diastolic dysfunction and HFpEF.

### Valve disease

Significant valve disease was identified in 16 (12.1%) in the AC + MRT group, 5 (4.4%) in the AC group and 3 (5.2%) in the controls. Moderate or severe mitral insufficiency was found in 4 (3.0%) in the AC + MRT group, 2 (1.8%) in the AC group and 2 (3.4%) in the controls. Mitral stenosis was found in 2 (1.5%) in the AC + MRT group, 1 (0.9%) in the AC group and in none of the controls. Aortic stenosis was found in 7 (5.3%) in the AC + MRT group, 3 (2.6%) in the AC group and 1 (1.7%) in the controls. Moderate or severe aortic insufficiency was found in 6 (4.5%) in the AC + MRT group and none in the AC group or in the controls. Participants with significant valve disease were older than those without (53.3 years vs. 45.8 years, *p* < 0.01). When adjusted for sex, age and treatment center, the AC + MRT group had an OR of significant valve disease of 5.0 (1.5–15.8, *p* < 0.01) compared to the AC-group.

### Dose-dependent effects on cardiac function

Total cumulative anthracycline dose showed a negative correlation with TAPSE, which remained significant when adjusted for sex, age, and treatment center (*R*=-0.15, *p* < 0.05). No such correlation was found for total anthracycline dose and GLS or EF.

Total mediastinal radiation dose showed a negative correlation with GLS, which remained significant when adjusted for sex, age, and treatment center (*R*=-0.18, *p* < 0.01). No such correlation was found for total mediastinal radiation dose and EF or TAPSE.

### Coronary artery disease

Established coronary artery disease (CAD) was reported by 9 (7%) in the AC + MRT group, 4 (4%) in the AC group and in 1 (2%) control. Of these, prior myocardial infarction was reported in 5 (4%) in the AC + MRT group, 4 (4%) in the AC group and in none of the controls. After adjusting for sex and age, the differences between groups were not significant.

## Discussion

Traditional treatments for HL have been associated with a high risk of developing CVD [3]. Reducing adverse cardiac effects, without compromising the efficacy of cancer treatment, has been an important area of research [6, 18]. Our findings suggests that steps taken to reduce cancer therapy-related adverse cardiac effects in the treatment of HL have been successful. Although adverse cardiac effects have not been eliminated, the average HLS treated after the late 1990s is now less likely to experience CVD than those treated in preceding decades.

### Cardiac function and morphology

We found no difference between HLS and controls regarding EF, the most commonly used measure of LV function. While a drop in EF is a late sign of cancer therapy-related cardiac dysfunction (CTRCD), it is closely associated to overt symptoms of heart failure and is an important predictor of prognosis [14]. Without reduced EF, asymptomatic CTRCD can never be classified as more than mild, according to ESC guidelines on cardio-oncology [19]. The lack of adverse effects about 16 years after modern treatment of HL on EF is thus a positive development.

While adverse cardiac effects have been mitigated, a substantial number of HLS in our study have a GLS outside the normal range, in fact, the risk for borderline or impaired GLS was more than three-fold compared to controls. It did not seem to matter whether the HLS had received MRT, as the prevalence of borderline or low GLS did not differ between treatment groups. Average GLS was lower in HLS receiving MRT, suggesting that although not more prevalent, those with impaired GLS after MRT had slightly worse function. It should be noted, however, that in most of these cases, GLS was borderline low, and although there is some evidence that this is associated with later adverse cardiac events, the clinical significance on prognosis is debatable [20].

Tsai et al. performed an echocardiographic study in 2011 which assessed HLS treated with MRT (mean 41 Gy) with or without anthracyclines between 1980 and 1988 [21] Unlike the HLS in the present study, these patients had worse cardiac function and outcomes. Patients treated with both anthracyclines and radiation, usually mantle field radiation, had an average GLS of 16.1 ± 1.9%. Although they were slightly older (51 ± 9 years) and had slightly longer observation time (22 ± 2 years) than in the present study, the difference is substantial. CTRCD from treatment with anthracyclines is highly dependent on cumulative dose, with little CTRCD below 200 mg/m^2^, and a steep increase in CTRCD above 400 mg/m^2^ [22]. However, even lower cumulative doses of anthracyclines can amplify the CTRCD of adjuvant radiation therapy. This has previously also been shown in survivors of breast cancer [23].

Even though a direct comparison is not possible, our findings would suggest that HLS treated with both AC and MRT using contemporary treatment, with lower radiation doses and smaller fields, fared better with respect to cardiac function than those treated with only radiation therapy in the 2011 study (GLS − 19.3 vs. -17.5).

Morphology in the AC-group was very similar to controls, but this was not the case for patients in the AC + MRT-group. These patients had smaller chamber volumes, thinner posterior walls and lower LV mass compared to both controls and the AC-group. It would suggest at least a mild degree of cardiac atrophy. Similar observations have been made in prior studies both in cancer survivors treated with anthracyclines and those treated with mediastinal radiotheraphy [24–26]. This finding, while consistent across studies, is especially noteworthy as this group also have a higher prevalence of valvular disease. It is likely that the combination of radiotherapy and treatment with anthracyclines is particularly potent in reducing LV mass, as described by van der Velde et al. [26]. While the exact pathophysiology is not known, Jordan et al. have stipulated extracellular remodeling, apoptosis and atrophy secondary to damage sustained by cardiomyocytes [27].

### Valve disease

Perhaps the most convincing evidence of adverse effects of treatment was the prevalence of valve disease in HLS treated with MRT. Cardiac valves are particularly susceptible to radiation damage, a well-known issue in traditional treatment of both HL and left-sided breast cancer [28]. It is therefore not surprising that HLS who underwent MRT had more valve pathologies than both controls and those treated with chemotherapy alone. Even so, the prevalence is drastically lowered compared to that found in earlier studies. In 2003, Heidenreich et al. found a 29% prevalence of significant valve disease in irradiated HLS [25]. Even though the present cohort had a longer observation time, which is strongly correlated with increased prevalence of valve disease following radiation therapy, the prevalence of valve disease was less than half (12% vs. 29%) [29].

As with cardiac function, the improvement in accuracy of radiation treatment is likely to be an important contributing factor to the reduction in valve disease.

While earlier studies have found an increase in valve disease from treatment with anthracyclines, we found no evidence of such an effect [30]. As changes in cardiac morphology in heart failure patients can cause valve insufficiencies, it could be that earlier results were simply secondary to the development of heart failure and cardiac dilatation. This was first proposed by van Nimwegen et al., and our findings support that theory [1].

The lower total prevalence of valvulopathies also complicates analysis of specific valvulopathies, but like earlier findings, the aortic valve seems more vulnerable [25].

### Coronary artery disease, cardiovascular risk factors and biochemistry

We found no significant differences between treatment groups or between HLS and controls with regards to most cardiovascular risk factors. This is in line with what Florido et al. found at baseline in a recent study on CVD and risk factors in participants who would go on to either develop cancer or not develop cancer [31].There is strong evidence for an increased incidence of CAD in HLS, which is most pronounced in those treated with radiotherapy [32]. However, it is likely that this increase in risk is caused by direct endothelial damage, rather than indirectly through adversely modified risk factors. Nonetheless, the higher baseline risk for CAD would suggest that close monitoring and treatment of modifiable risk-factors is of particular importance in HLS [3].

Although not significant, there was a trend towards more CAD in the AC + MRT group compared to controls in the present study. As this study was not powered to assess rare outcomes, one should be careful about inferring too much from these findings. It would, however, at least suggest that CAD could still be an issue in HLS, particularly those treated with both AC and MRT. This is further supported by a recent study by Keegan et al., who found a substantial increase in risk for CVD in young patients treated for HL [33].

Both treatment groups had a higher NT-proBNP than controls, and the HLS treated with both AC and MRT had a higher NT-proBNP than those treated with AC alone. This would be expected from the higher prevalence of borderline and impaired GLS on echocardiography.

### Limitations

Cross sectional studies are susceptible to survival bias. Similarly, a paper recently assessed the risk of selection bias in longitudinal studies and found that those who for various reasons failed to participate in the follow-ups are at higher risk for death [34]. This study combine elements of both, but is likely less affected by these issues, as measurable clinical or subclinical long-term adverse cardiac effects on echocardiographic examinations are likely to precede critical illness and death by several years.

Although the HLS were matched with controls on several risk factors, there were significant differences between the two treatment groups. Patients treated with both AC and MRT were more likely to be male and more likely to be current or former smokers. Although a potential bias, this was adjusted for in all the major analyses.

While this was a large echocardiographic study, it is not powered to reliably assess rare outcomes. Although we did not find a statistically significant difference in CAD or heart failure, we cannot conclude that such a difference does not exist, nor that the HLS are not at increased risk for CVD later in life. Furthermore, the study is not powered to reliably assess specific valvulopathies.

## Conclusions

The treatment for HL has been proven to be highly effective but has been linked with significant late cardiac adverse effects. Advances in radiotherapy and optimization of chemotherapy since the late 1990s have attempted to remedy this, but the long-term cardiac impact has been uncertain. This is the first large echocardiographic study to assess the impact of these advances. Although still associated with slightly worse cardiac function and higher prevalence of valvular disease than controls, we find a lower rate of long-term cardiac adverse effects compared to earlier studies. It is likely that the advances in the treatment of HL have been successful, and that HLS treated today are less likely to experience CVD related to cancer treatment than those in preceding decades.

## Data Availability

No datasets were generated or analysed during the current study.

## Abbreviations

AC: Anthracycline-based chemotherapy
AC + MRT: Anthracycline-based chemotherapy + mediastinal radiotherapy
CVD: Cardiovascular disease
EACVI: European Association of Cardiovascular Imaging
EF: Ejection fraction
GLS: Global longitudinal strain
HFpEF: Heart failure with preserved ejection fraction
HFrEF: Heart failure with reduced ejection fraction
HL: Hodgkin’s lymphoma
HLS: Hodgkin’s lymphoma survivors
